# Characterization of Human Huntington's Disease Cell Model from Induced Pluripotent Stem Cells

**DOI:** 10.1371/currents.RRN1193

**Published:** 2010-10-28

**Authors:** Ningzhe Zhang, Mahru C. An, Daniel Montoro, Lisa M. Ellerby

**Affiliations:** ^*^Buck Institute for Age Research and ^‡^Stanford University Medical School

## Abstract

Huntington’s disease (HD) is a dominantly inherited neurodegenerative disease caused by a CAG repeat expansion in the first exon of the gene Huntingtin (Htt). A dramatic pathological change in HD is the massive loss of striatal neurons as the disease progresses. A useful advance in HD would be the generation of a human-derived HD model to use for drug screening and understanding mechanisms of HD. We utilized the recently established human iPS cell line derived from HD patient fibroblasts to derive neuronal precursors and human striatal neurons. To achieve this goal, the differentiation of the HD-iPS cells into striatal fate required several steps. First, we generated nestin+/PAX6+/SOX1+/OCT4- neural stem cells (NSCs) from HD-iPS cells using the method of embryoid body formation. HD-NSCs were then subjected to a differentiation condition combining morphogens and neurotrophins to induce striatal lineage commitment. Striatal neuronal precursors/immature neurons stained with β-III tubulin, calbindin and GABA but not DARPP-32 (dopamine- and cyclic AMP-regulated phosphoprotein, Mr = 32,000) were produced in this step. Finally, maturation and terminal differentiation of the striatal neuronal precursors/immature neurons resulted in striatal neurons expressing markers like DARPP-32. The HD-iPS cells derived striatal neurons and neuronal precursors contain the same CAG expansion as the mutation in the HD patient from whom the iPS cell line was established. Moreover, the HD-NSCs showed enhanced caspase activity upon growth factor deprivation compared to normal NSCs (from iPS or H9 NSCs). Therefore, these differentiated cells may produce a human HD cell model useful in the study of HD mechanisms and drug screening.

## Introduction

The Huntington’s disease (HD) is a dominantly inherited neurodegenerative disease caused by a polyglutamine expansion in the N-terminus of the huntingtin protein.  Greater than 36-38 CAG repeats in huntingtin will cause HD and longer CAG repeat lengths correlate with earlier onset of the disease [1]
[2].  The most dramatic pathological change in HD brain is the massive loss of medium spiny neurons (MSNs) in the striatum and loss of neurons in the cortex.  The disease results in chorea, dementia and eventually death.  There are numerous mechanisms proposed for HD including proteolysis to generate toxic N-terminal fragments, alterations in vesicular trafficking, mitochondrial function and transcriptional dysregulation [3]
[4].

Currently there is no cure for HD.  Treatments alleviate symptoms but do not prevent or delay disease progression [5].  Studies aimed at understanding the cause of MSN cell loss in HD and efforts to develop new therapeutics would benefit from the generation of human medium spiny neurons carrying the genetic mutation for Htt.  Recent technology to reprogram patient specific skin fibroblasts to a pluripotent state offers this possibility [6].  Multiple high throughput screenings are also ongoing in search for potential drug candidates using cell culture models derived from overexpression of human Htt or mouse knockin cells [7].  Generation of a human and patient specific HD cell model would offer a number of advantages in our search for targets and therapeutics for HD including (1) accounting of genetic factors in each patient’s cell type, (2) generation of different cell types to understand selective vulnerability, (3) large supply human and patient specific primary cells,  (4) ability to recapitulate HD disease phenotype and (5) a possible cell therapy that avoids immune rejection.

We have utilized a recently established HD-specific induced pluripotent stem cell (iPSC) line to generate a human HD cell model with a CAG expansion mutation in the endogenous huntingtin gene.  The HD-specific iPSC (HD-iPSC) line was originally derived from a HD patient with a 72-repeat CAG tract by Park *et al*
[8].  Although mutant huntingtin is already expressed in HD-iPSCs (unpublished data), neuronal cells from the HD-iPSCs would more closely mimic the affected cells in HD.  Here we show that we can differentiate the HD specific neural stem cells (HD-NSCs) into neurons with striatal characteristics using a modified protocol based on work of Aubry *et al*  [9].  The HD-iPSC-derived neurons contain the same expanded CAG repeat number as the original HD-iPSC line and the HD patient fibroblasts from which this HD-iPSC line was generated. One important feature of HD pathology is the elevation of caspase-3/7 activity.  When we measure caspase-3/7 activity of the HD-NSCs and wild-type NSCs (WT-NSCs from normal iPS or H9 ESCs) 24 hours after withdrawal of growth factors, the HD-NSCs but not WT-NSCs showed enhanced caspase activity. Our results indicate that the HD-NSCs might serve as a human HD cell model with endogenous CAG expansion suitable for HD mechanistic studies and drug screenings.

## Results


**1. HD-iPSCs maintain ES cell markers after extended culture**


We cultured the HD-iPSCs and normal iPSCs ( from Dr. George Daley) in the same conditions of conventional human ESCs and immunostained these cells periodically to ensure expression of hESC markers.  Previous work demonstrated that the HD-iPSCs were pluripotent and capable of multilineage differentiation (8) . After extended culture of the HD-iPSCs on either MEF feeder cells or  Matrigel (around 60 passages) these cells still stained positive for classic hESC markers including OCT4, NANOG, SOX2 and SSEA4 [Figure 1], indicating  HD-iPSCs retained stem cell markers during passaging.  We did note that the normal-iPSCs and H9 ESCs when compared to HD-iPSCs had distinct levels of ERK activation in response to bFGF and the level of ERK was significantly dampened in HD-iPSCs.  This is consistent with previous reports in the literature of HD cell culture models of altered ERK activation [10] [Figure 2]. 

**Figure d20e110:**
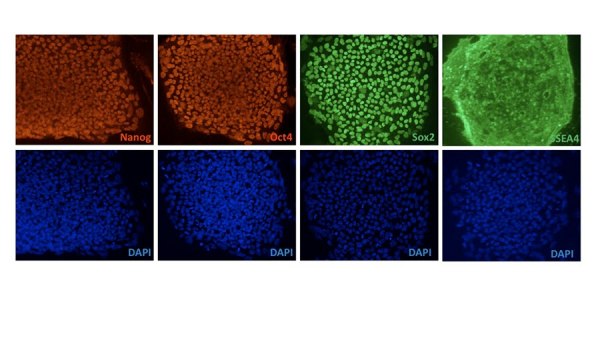

**Figure 1.  HD-iPS cells express classic ES cell markers. **HD-iPS cells were cultured in the same conditions as human ES cells and immunostained for classic ES cell markers OCT4, NANOG, SOX2 and SSEA4.

**Figure d20e117:**
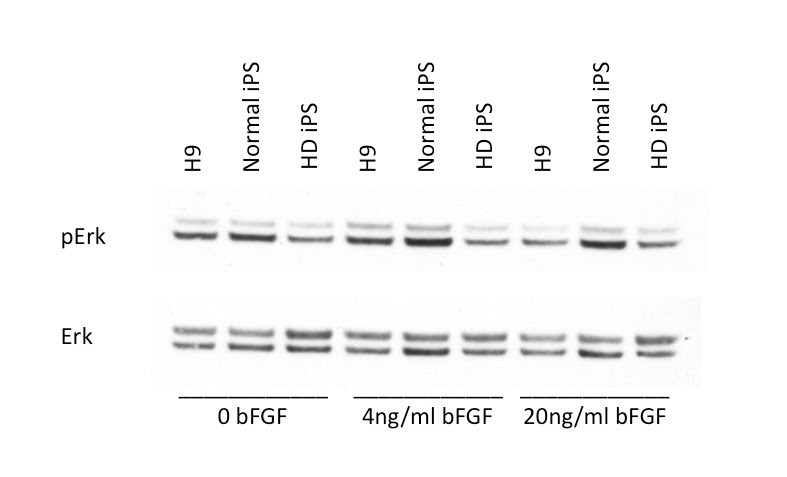


**Figure 2.  HD-iPSCs have altered signaling pathways when compared to control iPS. **HD-iPSCs, normal iPSCs and H9 cells were treated with 0, 4 or 20 ng/ml bFGF 24h after previous feeding. 30 min after bFGF treatment cells were harvested for protein lysates. Western blotting was performed to detect phospho-p44/42 (ERK1/2) (Thr202/Tyr204) as well as total p44/42 (ERK1/2) in these cells. The two bands represent p44 and p42 (either phosphor form or total protein) respectively.


**2. HD-iPSCs can be differentiated into HD-NSCs**


We differentiated HD-iPSCs into neural lineages with a previously established method that utilizes the formation of embryoid body (EB) intermediates. Neural rosettes appeared after attachment of EBs onto poly-ornithine/laminin (pO/L) coated surfaces in neural differentiation medium supplemented with bFGF.  We manually isolated the rosette cells, disrupted rosettes into smaller pieces mechanically and plated these rosette cells on pO/L coated surfaces in neural proliferation medium supplemented with bFGF.  After the first passage with 0.05% trypsin these monolayer cells were cultured routinely as NSCs. The yield for this step was greater than 95%.  Immunofluorescent staining showed that these cells expressed NSC markers Nestin, SOX1 and PAX6 but not the ESC marker OCT4 [Figure 3].

**Figure d20e131:**
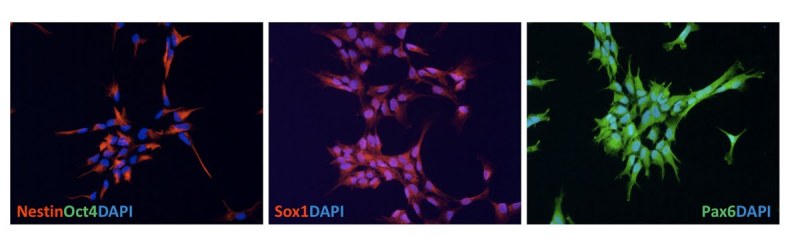


**Figure 3.  HD-NSCs derived from HD-iPS cells. **The HD-NSCs derived from HD-iPS cells immunostained positive for NSC markers Nestin, SOX1 and PAX6 while negatively for ES cell marker OCT4.


**3. HD-NSCs can be differentiated into striatal neurons**


Next we further developed conditions to produce striatal neurons from the HD-NSCs. We modified a previously published protocol based on work of Aubry et al. [9] by treating the HD-NSCs directly with SHH, DKK1 and BDNF. In addition, we included the ROCK inhibitor Y27632 in the differentiation condition to promote cell survival. After 8-10 days in SHH, DKK1, BDNF and Y27632 (Stage 1), cells were further differentiated with BDNF, cAMP, valproic acid and Y27632 for an additional 1-3 days (Stage 2). At Stage 1, cells already expressed GABAergic neuron marker GABA, some even stained with striatal neuron marker calbindin, but no MSN specific marker DARPP-32 positive cells were observed [Figure 4A]. At stage 2 some cells started to express DARPP-32 [Figure 4B]. This differentiation experiment demonstrated that these HD-NSCs were capable of giving rise to striatal neurons, which may also be used as an HD cell model.  The yield of DARPP-32 neurons was approximately 10%. Since we induced striatal differentiation stepwise, it is not easy to estimate overall percentage of striatal neurons generated from original iPS cells. After EB attachment rosettes represented a small portion of total cells (approximately 20%). However these rosettes cells are enriched NSCs (greater than 95% based on Nestin staining) and they can be passaged for over 20 passages and still stain positive for NSC markers Nestin, Sox1 and Pax6. So the amount of NSCs was not a limiting factor. The yield from NSCs to DARPP32 positive neurons was low. Higher purity DARPP32 neurons may require introducing a striatal specific reporter and cell sorting.

**Figure d20e150:**
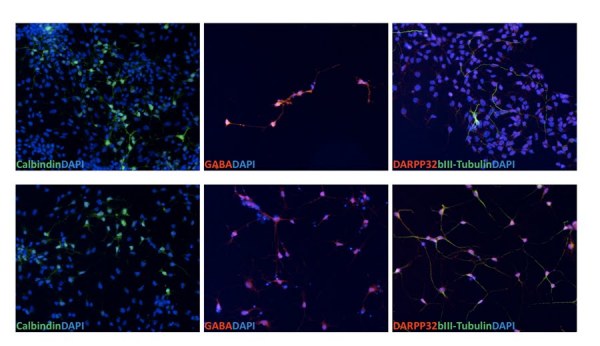


**Figure 4.  Striatal neurons derived from HD-iPS cells. A **(Stage 1) Immature striatal neurons express neuronal marker βIII-tubulin (Tuj1), GABAergic neuron marker GABA and striatal marker calbindin but not medium spiny neuron (MSN) marker DARPP-32. **B** (Stage 2) Mature striatal neurons express DARPP32 as well as well as βIII-tubulin, GABA and Calbindin.


**4. The endogenous HD mutation persisted in all cell types**


The CAG expansion mutation of HD is sometimes unstable and may contract or expand spontaneously [1]
[2] . To make sure our HD cell model contained the same length of CAG repeats as parental cells, we performed a PCR to amplify the CAG trinucleotide tract. The PCR result showed that all the cells derived from HD-iPSCs (line HD-iPS-4), including HD-NSCs and striatal differentiated neurons, had 72 CAG repeats, same as the HD patient fibroblasts from which the HD-iPSCs were generated [Figure 5]. In contrast normal iPS cells (line F-iPS-5 from Dr. George Daley) only contained alleles with normal CAG repeats. The stability of the CAG trinucleotide repeats in our HD-NSCs and striatal differentiated neurons ensured the consistency and reproducibility for the use of these cells as HD model. Note that iPS (line HD-iPS-1) was determined to have normal repeat length (data not shown).

**Figure d20e173:**
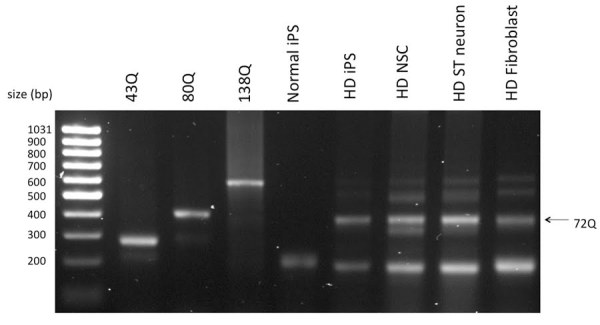


**Figure 5. All cells derived from HD-iPS cells contain the same CAG expansion mutation as the parental HD-fibroblasts. **Total DNA was extracted from normal iPS cells, HD-iPS cells, NSC and striatal neurons derived from HD-iPS cells, as well as HD-fibroblasts from which the HD-iPS cells were generated. A PCR reaction amplifying the CAG trinucleotide tract containing *huntingtin* sequence was performed with total DNA samples. cDNAs containing known number of CAG repeats were also included. Unlike Normal iPS cells, HD-iPS cells and cells derived from HD-iPS cells have the exact number of CAG repeat as the HD-Fibroblasts.


**5. Enhanced caspase activity in growth factor deprived HD-NSCs when compared to WT-NSCs**


One of the most important applications of a HD cell model is to screen for therapeutic compounds with cellular assays. One such assay utilized in the field is the measurement of caspase activity.  Caspase activity is elevated in HD cell culture models, mouse models and postmortem tissue when compared to controls (11) .  Because caspase cleavage of huntingtin is an important step to generate toxic fragments, the activity of caspase reflects Htt-mediated cellular toxicity. To test if our human HD iPSC derived model is suitable for assay based drug screenings, we measured caspase activity in HD-NSCs and WT-NSCs (NSCs derived from normal H9 ES cells[11] using the same EB method or from iPSC with normal HD repeat length) after growth factor withdrawal for 24h. In WT-NSCs, 24h of growth factor withdrawal did not affect caspase-3/7 activity significantly. However, in HD-NSCs caspase-3/7 activity was stimulated under the same condition [Figure 6].  This result demonstrates the potential use of this human HD cell model in drug screenings.

**Figure d20e194:**
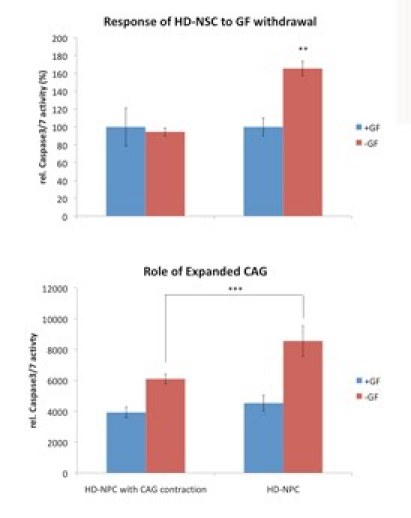


**Figure 6. Higher cellular toxicity of HD-NSCs compared to normal NSCs. **Both HD and normal NSCs were subject to a caspase-3/7 activity assay 24h after growth factor withdrawal. HD-NSCs showed elevated toxicity while normal NSCs did not. (** P<0.01 and *** P<0.001 in paired t-test)  The upper panel the normal NSCs were derived from human H9 embryonic stem cells.  The lower panel the NSCs were derived from iPS with normal CAG repeat length (HD-iPS line 1) .

## Discussion 

We have demonstrated that HD patient-specific pluripotent stem cells can be cultured over multiple passages without losing CAG repeat length or pluripotent markers such as SSEA-4, NANOG, OCT4 and SOX2.  These markers are absent in the fibroblasts in which the iPSCs were derived (data not shown).  As might be expected, we do detect altered signalling when comparing the normal iPSCs to the HD iPSCs.  In this case, we detect altered ERK phosphorylation in resting cells and in response to bFGF.

A characteristic property of pluripotent cells is their ability, when plated in suspension culture, to form embryoid bodies (EB).  We found both the normal and HD iPSCs formed EBs.  The hope is that HD iPSCs can be differentiated into the disease relevant cell types. We found that from EBs, HD iPSCs can be differentiated into neuronal precursor cells in high yield.  These HD-NSCs expressed markers nestin, SOX1 and PAX6.  The HD NSCs could be further differentiated into cells expressing GABAergic neuron marker GABA and a subset of these stained with neuron marker calbindin.  Further differentiation yielded DARPP-32 positive neurons.  Currently we are optimizing the yield of the DARPP-32 positive cells. However, even this mixed culture may yield clues to cell selective neuronal vulnerability in HD.

Finally, we found that normal NSCs when compared to HD NSCs have altered levels of caspase activity during serum withdrawal. This phenotype could be utilized for screening of therapeutic compounds or to optimize differentiation procedures. Although the possible differences in ERK activation and caspase induction are promising they will need to be confirmed in multiple HD iPS from distinct patient fibroblasts.

 Many insights into molecular pathways in HD come from analysis of post-morterm HD tissue or HD mouse models.  With the opportunity to utilized patient specific iPS cells, new tools will be generated to understand mechanisms of HD.

## Materials and Methods

### 
**Cell culture**


All cell culture reagents were from Invitrogen unless otherwise mentioned. HD-iPSCs were cultured like ES cells on either Matrigel (BD) or irradiation inactivated mouse embryonic fibroblasts (MEFs). When HD-iPSCs were grown on MEFs, the ES culture medium was knockout DMEM/F12 supplemented with 20% knockout serum replacement, 2.48mM L-glutamine, 1X nonessential amino acid, 15.4mM HEPES, 50μM β-mercaptoethanol, 100U/ml penicillin, 100μg/ml streptomycin (Cellgro) and 4ng/ml bFGF (Peprotech). When HD-iPSCs were grown on Matrigel, the ES medium conditioned by MEFs was used. The HD-iPSCs were regularly passaged with collagenase. HD-NSCs and WT-NSCs were cultured on plates coated with 20μg/ml poly-ornithine (Sigma) for 1h at 37^o^C followed by 5μg/ml mouse laminin (Sigma) for 1h at 37^o^C. The neural proliferation medium for NSCs was ENStem-A Neural Expansion Medium (Millipore, NeuroBasal medium with 1X B27, 10ng/ml LIF) supplemented with 2mM L-glutamine, 100U/ml penicillin, 100μg/ml streptomycin, and 25ng/ml bFGF. NSCs were regularly passaged with Accutase (Sigma). 


**Western blotting**


HD-iPSCs, normal iPSCs or H9 cells cultured on Matrigel were fed with bFGF containing MEF conditioned medium daily. On the day of experiment, without feeding cells with fresh medium different doses of bFGF (4ng/ml or 20ng/ml) were added to these cells which had not been fed for 24h. 30 min after bFGF treatment cells were scraped off, pelleted and washed once with DPBS (Cellgro). Cell pellets were lyzed by sonication in mammalian protein extraction reagent (M-PER, from Thermo Scientific) containing protease inhibitors (one Complete mini tablet per 10ml, Roche) and 1% phosphatase inhibitor cocktail set II (Calbiochem). Protein concentration was measured with Pierce BCA protein assay kit (Thermo Scientific) to ensure equal sample loading. Protein samples were run on 4-12% bis-tris gel (Invitrogen), transferred to nitrocellulose membrane (Whatman), probed with anti-phospho-p44/42 (ERK1/2) (Thr202/Tyr204) antibody (Cell Signaling) and reprobed with anti-p44/42 (ERK1/2) antibody (Cell Signaling). 


**NSC and striatal differentiation**


HD-NSCs were derived from HD-iPSCs with EB method. Briefly HD-iPSCs were passaged with collagenase and cell clumps were cultured in a low attachment petri-dish (Kord-Valmark) in ES medium without bFGF. Medium was replaced every 2 days and at each time of medium change 25% more ES medium was replaced by EB differentiation medium (DMEM supplemented with 20% fetal bovine serum, 1X nonessential amino acid, 50μM β-mercaptoethanol, 100U/ml penicillin and 100μg/ml streptomycin). After 8 days the medium was 100% EB differentiation medium. After 10 days the EBs in suspension were attached onto pO/L coated plates in neural differentiation medium (DMEM/F12 supplemented with 1X N2, 100U/ml penicillin and 100μg/ml streptomycin) with 25ng/ml bFGF. Medium was replaced every 2 days. After 10-12 days rosettes were manually picked, triturated by P1000 tip and plated on pO/L coated plates in neural proliferation medium. The first passage was performed with 0.05% Trypsin and the following passages were done with Accutase (Sigma). WT-NSCs were derived from H9 human ESCs by the same procedure. The striatal differentiation of HD-NSCs was induced by changing neural proliferation medium to neural differentiation medium supplemented with 250ng/ml SHH (R&D Systems), 100ng/ml DKK1 (R&D Systems), 20ng/ml BDNF (Peprotech) and 10μM Y27632 (Calbiochem). After 8-10 days in the condition above (Stage 1), cells were exposed to 0.5mM dibutryl-cyclic AMP (Sigma), 0.5μM valpromide (Alfa Aesar), 20ng/ml BDNF and 10μM Y27632 for an additional 1-3 days (Stage 2).


**CAG repeats PCR**


Total DNA was extracted from different cell samples with DNeasy kit (Qiagen) according to manufacturer’s instructions. The primers used to amplify the CAG trinucleotide containing fragment are: Forward 5’-CCT TCG AGT CCC TCA AGT CCT TC-3’, Reverse 5’-GGC GGG GGC GGC TGC GGC TGA G-3’.


**Caspase-3/7 activity assay**


The caspase activity assay was performed with Apo3 HTS kit (Cell Technology). Either HD-NSCs or WT-NSCs were grown in 24 well plates. For growth factor deprived samples cells were first washed once with neural proliferation medium without LIF and bFGF, then cultured in this growth factor free medium for 24h. After 24h of growth factor withdrawal, medium was removed and 150μl 1X lysis buffer was added into each well. The plate were placed on an orbital shaker at 700 rpm for 10 min. Then, 30μl of cell lysate was dispensed into one well of a 96 well plate in triplicate for each sample. 70μl of substrate mix (1X lysis buffer with 1X Apo3 HTS Caspase3/7 detection reagent and 20mM DTT) was added into each well and the plate was shaken at 700rpm for 30s. Subsequently the plate was loaded on Fusion-Alpha Universal Microplate Analyzer (Perkin Elmer) for the fluorescence based reading (Ex: 485nm, Em: 530nm). For each sample 10μl of lysate was dispensed into another 96 well plate in triplicate for protein concentration measurement with Pierce BCA protein assay kit. The caspase activity was normalized against protein concentration for each sample.

## Acknowledgements 

We thank Dr. Daley for providing us the normal and HD-iPSCs. Correspondence should be sent to lellerby@buckinstitute.org, Buck Institute for Age Research, Novato, CA 94945

## Funding

 This work was funded by the Buck Institute for Age Research and NIH T32 training grant AG000266 (MCA).  

## Competing Interests

The authors have declared that no competing interests exist.

## References

 1.            Huntingtons Disease Collaborative Research Group 1993. A novel gene containing a trinucleotide repeat that is expanded and unstable on Huntington's disease chromosomes. The Huntington's Disease Collaborative Research Group. *Cell* 72:971-983.

2.            Duyao, M., Ambrose, C., Myers, R., Novelletto, A., Persichetti, F., Frontali, M., Folstein, S., Ross, C., Franz, M., Abbott, M., et al. 1993. Trinucleotide repeat length instability and age of onset in Huntington's disease. *Nat Genet* 4:387-392.

3.            Wellington, C.L., Ellerby, L.M., Hackam, A.S., Margolis, R.L., Trifiro, M.A., Singaraja, R., McCutcheon, K., Salvesen, G.S., Propp, S.S., Bromm, M., et al. 1998. Caspase cleavage of gene products associated with triplet expansion disorders generates truncated fragments containing the polyglutamine tract. *J Biol Chem* 273:9158-9167.

4.            Wellington, C.L., Singaraja, R., Ellerby, L., Savill, J., Roy, S., Leavitt, B., Cattaneo, E., Hackam, A., Sharp, A., Thornberry, N., et al. 2000. Inhibiting caspase cleavage of huntingtin reduces toxicity and aggregate formation in neuronal and nonneuronal cells. *J Biol Chem* 275:19831-19838.

5.            Imarisio, S., Carmichael, J., Korolchuk, V., Chen, C.W., Saiki, S., Rose, C., Krishna, G., Davies, J.E., Ttofi, E., Underwood, B.R., et al. 2008. Huntington's disease: from pathology and genetics to potential therapies. *Biochem J* 412:191-209.

6.            Takahashi, K., and Yamanaka, S. 2006. Induction of pluripotent stem cells from mouse embryonic and adult fibroblast cultures by defined factors. *Cell* 126:663-676.

7.            Fecke, W., Gianfriddo, M., Gaviraghi, G., Terstappen, G.C., and Heitz, F. 2009. Small molecule drug discovery for Huntington's Disease. *Drug Discov Today* 14:453-464.

8.            Park, I.H., Arora, N., Huo, H., Maherali, N., Ahfeldt, T., Shimamura, A., Lensch, M.W., Cowan, C., Hochedlinger, K., and Daley, G.Q. 2008. Disease-specific induced pluripotent stem cells. *Cell* 134:877-886.

9.            Aubry, L., Bugi, A., Lefort, N., Rousseau, F., Peschanski, M., and Perrier, A.L. 2008. Striatal progenitors derived from human ES cells mature into DARPP32 neurons in vitro and in quinolinic acid-lesioned rats. *Proc Natl Acad Sci U S A* 105:16707-16712.

10.            Apostol BL, Illes K, Pallos J, Bodai L, Wu J, Strand A, Schweitzer ES, Olson JM, Kazantsev A, Marsh JL, Thompson LM. 2005 Mutant huntingtin alters MAPK signaling pathways in PC12 and striatal cells: ERK1/2 protects against mutant huntingtin-associated toxicity. *Hum Mol Genet* 15, 273-285.

11.            Hermel, E., Gafni, J., Propp, S.S., Leavitt, B.R., Wellington, C.L., Young, J.E., Hackam, A.S., Logvinova, A.V., Peel, A.L., Chen, S.F., et al. 2004. Specific caspase interactions and amplification are involved in selective neuronal vulnerability in Huntington's disease. *Cell Death Differ* 11:424-438.

12. Thomson, J.A., Itskovitz-Eldor, Shapiro, S.S., Waknitz, M.A., Swiergiel, J.J., Marshall, V.S., Jones, J.M. 1998 Embryonic stem cell lines derived from human blastocysts. Science 282, 1145-1147.
